# Hybrid Frenkel–Wannier excitons facilitate ultrafast energy transfer at a 2D–organic interface

**DOI:** 10.1038/s41567-025-03075-5

**Published:** 2025-10-29

**Authors:** Wiebke Bennecke, Ignacio Gonzalez Oliva, Jan Philipp Bange, Paul Werner, David Schmitt, Marco Merboldt, Anna M. Seiler, Kenji Watanabe, Takashi Taniguchi, Daniel Steil, R. Thomas Weitz, Peter Puschnig, Claudia Draxl, G. S. Matthijs Jansen, Marcel Reutzel, Stefan Mathias

**Affiliations:** 1 I. Physikalisches Institut, Georg-August-Universität Göttingen, Göttingen, Germany; 2 Physics Department, CSMB, Humboldt-Universität zu Berlin, Berlin, Germany; 3 Research Center for Electronic and Optical Materials, National Institute for Materials Science, Tsukuba, Japan; 4 Research Center for Materials Nanoarchitectonics, National Institute for Materials Science, Tsukuba, Japan; 5 International Center for Advanced Studies of Energy Conversion, University of Göttingen, Göttingen, Germany; 6 Institute of Physics, NAWI Graz, University of Graz, Graz, Austria; 7 European Theoretical Spectroscopic Facility, Berlin, Germany; 8 Fachbereich Physik, Philipps-Universität Marburg, Marburg, Germany; 9 mar.quest, Marburg Center for Quantum Materials and Sustainable Technologies, Marburg, Germany

**Keywords:** Surfaces, interfaces and thin films, Two-dimensional materials

## Abstract

Two-dimensional transition metal dichalcogenides and organic semiconductors have emerged as promising material platforms for optoelectronic devices. Combining the two is predicted to yield emergent properties while retaining the advantages of each. In organic semiconductors, the optoelectronic response is typically dominated by localized Frenkel-type excitons, whereas transition metal dichalcogenides host delocalized Wannier-type excitons. However, much less is known about the characteristics of excitons at hybrid interfaces between these materials, which determine the possible energy- and charge-transfer pathways. Here we identify a hybrid exciton at one such interface using ultrafast momentum microscopy and many-body perturbation theory. We show that this hybrid exciton, formed predominantly via resonant Förster energy transfer, has both Frenkel- and Wannier-type contributions: intralayer electron–hole transitions within the organic semiconductor layer and interlayer transitions across the interface give rise to an exciton wavefunction with mixed character. This work advances our understanding of charge and energy transfer processes across 2D–organic heterostructures.

## Abstract (web summary)

A hybrid exciton is observed at the interface between an organic semiconductor and a transition metal dichalcogenide. This suggests engineering the exciton wavefunction can lead to efficient charge and energy transfer processes in such structures.

## Main

Hybrid Frenkel–Wannier excitons are Coulomb-bound electron–hole pairs that join the unique optical properties of Frenkel excitons with the wavefunction delocalization of Wannier excitons^1–4^. Such excitons have been observed to occur at the interface of organic–inorganic heterostructures^1^ and in organic–inorganic microcavities^2^. Moreover, hybrid Frenkel–Wannier excitons have been proposed to mediate charge and energy transport^5–9^ and to be a promising platform for studying many-body exciton physics^10^ as well as for nonlinear optical applications^3^. However, the experimental characterization of the fundamental nature of such Frenkel–Wannier excitons has remained inaccessible so far. Much is unknown about the electronic composition and the spatial characteristics of the interfacial excitonic wavefunctions.

A highly promising platform for the realization of hybrid Frenkel–Wannier excitons is given by heterostructures of two-dimensional (2D) transition metal dichalcogenides (TMDs) combined with organic semiconductors (OSCs). Both these material classes are known for reduced electronic screening that leads to the formation of strongly Coulomb-bound excitons. In OSCs, predominantly Frenkel-type and charge-transfer excitons exist, which both derive their wavefunction from molecular orbitals and which are commonly restricted to only a single, or to just the neighbouring molecules^11–13^. Conversely, TMDs are known for hosting delocalized Wannier-type excitons whose wavefunctions are built up from Bloch states of the valence and conduction bands^14^. These individual properties make TMD/OSC heterostructures particularly promising for the realization of hybrid Frenkel–Wannier excitons, and it is predicted that both hybrid and charge transfer excitons can exist whose wavefunctions are composed of contributions of molecular orbitals of the OSC and valence/conduction band Bloch states of the TMD^15,16^. However, the spatial structure of their wavefunction, that is, whether the Frenkel or Wannier contributions to the wavefunction are more dominant, or if an exciton with both Frenkel and Wannier character can form and exist, remains largely unexplored. Moreover, experimental evidence for ultrafast charge- and energy-transfer processes is scarce.

Here, using femtosecond momentum microscopy^17^ as well as *G*_0_*W*_0_ quasiparticle band structure^18^ and Bethe–Salpeter equation (BSE)^19^ calculations, we identify and characterize a hybrid Frenkel–Wannier exciton in the prototype system of monolayer 3,4,9,10-perylenetetracarboxylic dianhydride (PTCDA) adsorbed on monolayer tungsten diselenide (WSe_2_). We chose this system because, on the inorganic side, the electronic band structure^20^, the energy landscape of excitons^14^ and the resulting ultrafast exciton dynamics^21–23^ are well characterized. Complementarily, on the organic side, PTCDA serves as a key model system for fabricating flat molecular layers of OSCs adsorbed on pristine surfaces^24^ and for the study of optical excitations^25,26^. This is an ideal setting to study the orbital contributions to all relevant optically bright- and dark-exciton wavefunctions. This includes the momentum-direct and momentum-indirect intralayer excitons in WSe_2_ and, intriguingly, the formation of a hybrid WSe_2_/PTCDA exciton. Our joint experimental and theoretical results show that this hybrid exciton’s wavefunction is a coherent superposition of intra- and interlayer contributions with Frenkel and Wannier character, respectively. Moreover, the orbital-resolved access to the exciton wavefunction combined with femtosecond time-resolution enables us to characterize the formation mechanism of the hybrid exciton. We show that, in response to the optical excitation of WSe_2_ A1s-excitons, exciton–phonon scattering and dominantly a Förster-type energy-transfer lead to the establishment of a steady state population between intralayer momentum-direct and momentum-indirect WSe_2_ excitons and the energetically most favourable hybrid exciton.

## TMD/OSC sample structure and single-particle energy level alignment

**Fig. 1:**
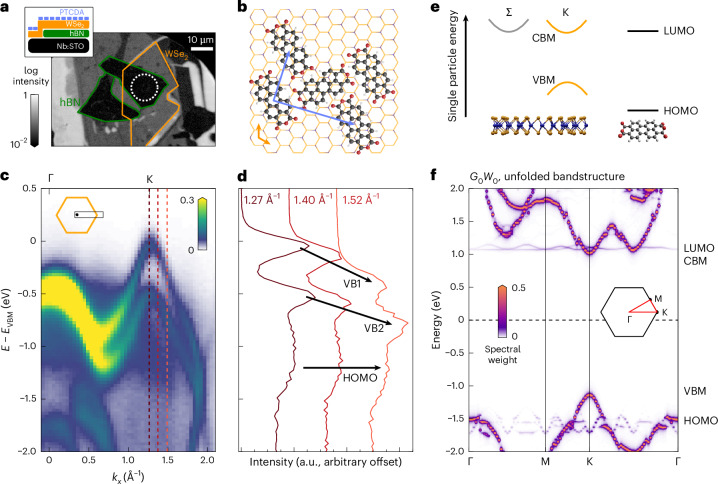
Sample layout and electronic structure of the hybrid WSe_2_/PTCDA heterostructure. **a**, A sketch of the layered sample structure and real-space photoemission image. The real-space region of interest addressed in the momentum-resolved photoemission measurement is marked by the dashed white circle, while the hBN flake and the WSe_2_ monolayer are indicated by coloured lines. **b**, Experimentally determined superstructure of PTCDA adsorbed on WSe_2_ monolayer (Extended Data Fig. 1). **c**, Energy–momentum cut of the static photoemission spectrum along the Γ–K direction of the WSe_2_/PTCDA heterostructure measured at 50 K. **d**, EDCs taken at the momenta indicated in **c**. The dispersive spin-split WSe_2_ bands (VB1 and VB2) and the non-dispersive HOMO level are marked by arrows. **e**, An overview of the type-I energy level alignment of the TMD/OSC heterostructure. The sketch is extracted from static photoemission spectroscopy (**c** and **d**), the *G*_0_*W*_0_ calculation (**f**) and STS experiments reported in refs. ^27,28^, which are in qualitative agreement. **f**, Unfolded single-particle energy landscape of the WSe_2_/PTCDA heterostructure as retrieved from the scissor-shifted *G*_0_*W*_0_ calculation in a 4 × 4 × 1 supercell (Extended Data Fig. 5c; Methods).

**Extended Data Fig. 1:**
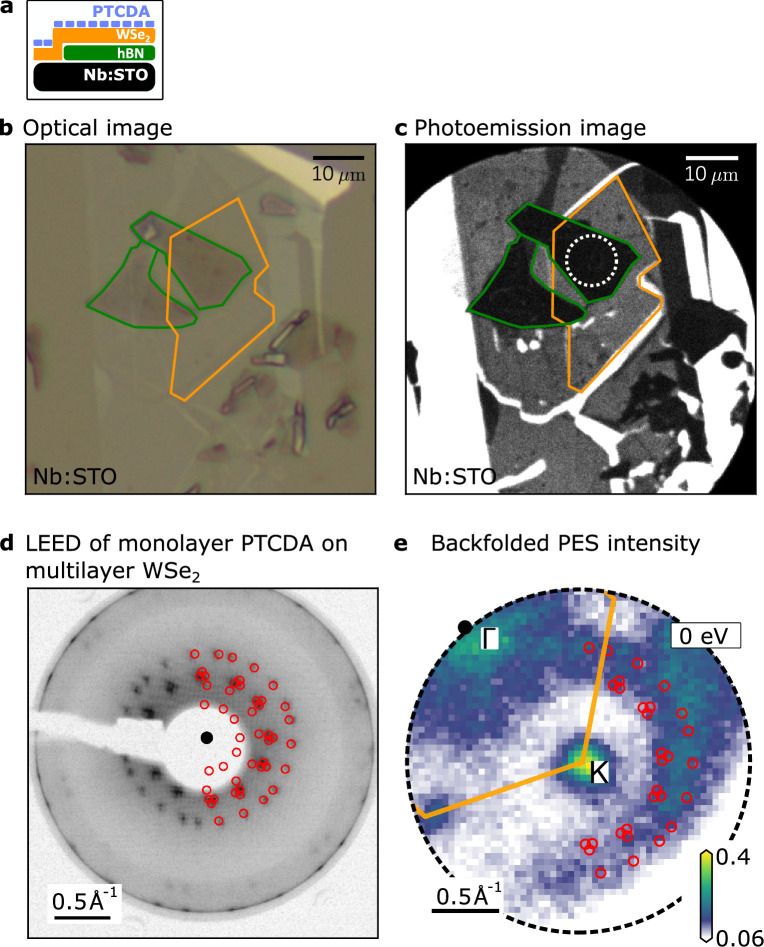
Analysis of the real-space structure of the WSe_2_/PTCDA heterostructure. **a** Sketch of the layered sample structure. **b**, **c** Optical microscope and photoemission real-space image of the sample before PTCDA evaporation. The different flakes are marked with the same colors as used in **a**. Note that the marked WSe_2_ area corresponds to an intact monolayer (without cracks) whereas the complete monolayer as seen by the contrast in **b** and **c** was larger. **d** LEED pattern of monolayer PTCDA on multilayer WSe_2_ recorded with a beam energy of 24 eV. The red circles correspond the superstructure defined by the matrix M = ((1.58,6.78), (4.39,1.32)). **e** The presence of a well-ordered PTCDA monolayer on the WSe_2_ monolayer is confirmed by the appearance of umklapp-scattering replicas of the WSe_2_ band structure, here shown for the valence band maximum at the K point (red circles). The replicas can be directly compared to the LEED pattern in **d**.

The WSe_2_ monolayer was fabricated by mechanical exfoliation and transferred on bulk hexagonal boron nitride (hBN) on a Nb:STO substrate (Fig. 1a; Methods). Subsequently, approximately a monolayer of PTCDA was evaporated under ultrahigh vacuum conditions. By analysing the Umklapp scattering of the photoemitted electrons at the molecular superstructure (Extended Data Fig. 1e), we verified that PTCDA adsorbs in an ordered herringbone structure with a 20.2 × 13 Å^2^ supercell (Fig. 1b).

**Extended Data Fig. 2:**
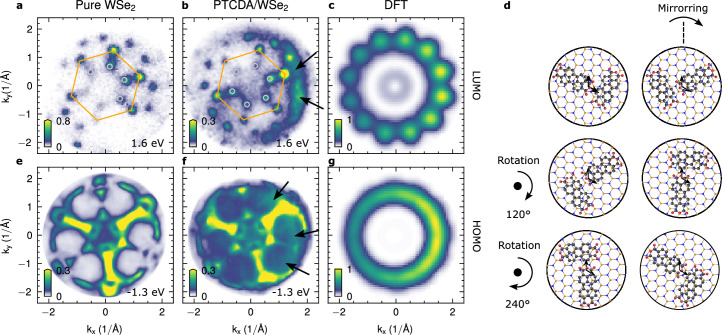
Direct comparison of ARPES data collected on pristine monolayer WSe_2_ and the WSe_2_/PTCDA heterostructure. **a,b,e,f** Momentum-maps collected at energies of the valence bands (bottom row) and at energies of excitonic photoemission signatures (top row). Photoemission signatures of the HOMO and the hX are labeled by arrows in **f** and **b**, respectively. **c**,**g** Simulated momentum maps from DFT calculations of the LUMO and HOMO of PTCDA using the plane wave model of photoemission^30^ and accounting for the herringbone structure and the different mirror and rotational domains shown in **d**. The DFT data are extracted from ref. ^66^.

To extract the energy-level alignment of this WSe_2_/PTCDA heterostructure, we start with static angle-resolved photoelectron spectroscopy (ARPES) experiments (26.5 eV extreme ultraviolet (EUV) photons, 20 fs, *p*-polarized). The energy- and momentum-resolved photoemission data and the momentum-filtered energy distribution curves (EDCs) are shown in Fig. 1c,d. We find clear signatures of the K valley WSe_2_ valence band maximum (VBM) (*E* − *E*_VBM_ = 0 eV) and additional spectral weight at an energy of *E* − *E*_VBM_ = −1.2 ± 0.1 eV that we identify with the highest occupied molecular orbital (HOMO) of PTCDA (see also momentum map in Extended Data Fig. 2f). We note that no signatures for hybridization of WSe_2_ valence states and PTCDA orbitals are observed. The energy level alignment of the WSe_2_ VBM and the PTCDA HOMO found here (Fig. 1e) is consistent with earlier scanning tunnelling spectroscopy (STS) experiments^27,28^, and, in addition, in qualitative agreement with our *G*_0_*W*_0_ calculations performed with the ‘exciting’ code^29^ (Fig. 1f; Methods). Combining all this information, we find that the single-particle energy-level alignment of the hybrid WSe_2_/PTCDA heterostructure is of type I, where the energies of both the lowest unoccupied and the highest occupied state are found in WSe_2_.

## Momentum-resolved characterization of excitons at the 2D–organic interface

**Fig. 2:**
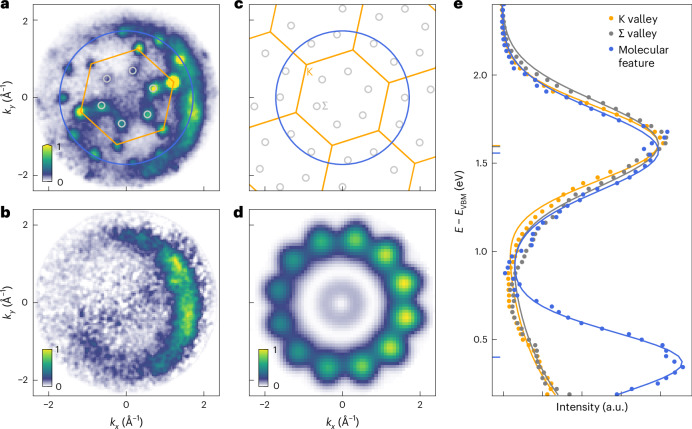
Energy- and momentum-resolved identification of the excitonic photoemission signatures. **a**,**b**,**e**, Energy-filtered momentum maps at *E* − *E*_VBM_ = 1.6 eV (**a**) and *E* − *E*_VBM_ = 0.4 eV (**b**) as well as the momentum-filtered EDCs (**e**) of excitonic photoemission signatures. The data were obtained by integrating over pump–probe delays from 100 to 500 fs, and applying a background subtraction using the non-negative matrix factorization formalism (Methods; Extended Data Fig. 3). In **a**, The WSe_2_ BZ is indicated by an orange hexagon. The K valleys lie at the corners of the hexagon. The Σ valleys are marked by grey circles. The blue circle with a radius of $\sqrt{{k}_{x}^{2}+{k}_{y}^{2}}$ ≈ 1.7 Å^−1^ corresponds the expected mean radius of the simulated momentum distribution of the LUMO of PTCDA shown in **d** (see also Extended Data Fig. 2). **c**, Extended zone scheme with the BZ marked in orange, the Σ points marked with grey circles and the molecular photoemission feature indicated by the blue circle. **d**, Simulated momentum map from DFT calculation of the LUMO of PTCDA using the plane wave model of photoemission^34^. **e**, The EDCs are filtered in momentum for the K (orange), Σ (grey) and molecular (blue) photoemission signatures (see Extended Data Fig. 4 for chosen region of interests) and fitted with a single or two Gaussian peaks (Methods). The resulting peak energies are marked with a horizontal bar in the plot, and the corresponding exciton energies ${{{E}}}_{{\rm{exc}}}^{i}$ are summarized in Extended Data Table 1.

**Extended Data Fig. 3:**
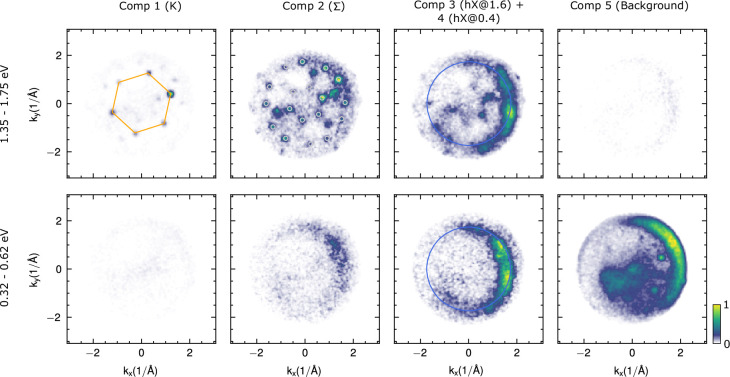
NMF component analysis of the ARPES data. Components 1, 2, 3, and 4 follow the temporal dynamics extracted for the K, *Σ*, hX@1.6eV, and hX@0.4eV photoemission features, respectively. Comp 5 corresponds to the static component, that is, the time-independent background which is subtracted from the raw data for the momentum maps and EDCs shown in Fig. 2. The photoemission features of the K-excitons, *Σ*-excitons, and the hX are marked by an orange hexagon, gray circles and a blue circle, respectively, in the components where most prominent. The energy regions of interest are chosen to be the same as the one shown in Fig. 2. Each component is normalized to the maximal value of the upper and lower panel.

The extension of the static ARPES experiment with a femtosecond pump–probe scheme is used to characterize the orbital contributions to optical excitations at the 2D–organic interface. The experimental conditions are chosen such that the driving photon energy of 1.7 eV (*s*-polarized, 40 fs) lies well below the lowest-energy optical excitation in PTCDA, which is ≿2 eV (ref. ^25^), that is, no PTCDA-only excitations can be created by the optical pump pulses. Instead, the laser pulses selectively excite the bright intralayer A1s-excitons in WSe_2_, which we label as K-excitons here (peak fluence 280 ± 20 μJ cm^−2^, exciton density (5.4 ± 1.0) × 10^12^ cm^−2^). All other excitons that are detected in the photoemission experiment must result from subsequent charge and energy transfer processes, as will be further discussed below. Figure 2a,b shows time-delay-integrated photoemission momentum maps collected in selected energy windows above the WSe_2_ valence bands (see the Methods and Extended Data Fig. 3 for background subtraction). In the (*k*_*x*_,*k*_*y*_)-momentum-resolved data, we find a rich intensity structure that provides direct evidence for the presence of excitons that are of pure WSe_2_ intralayer character, but also of excitons with distinct orbital contributions from the PTCDA layer.

**Extended Data Fig. 4:**
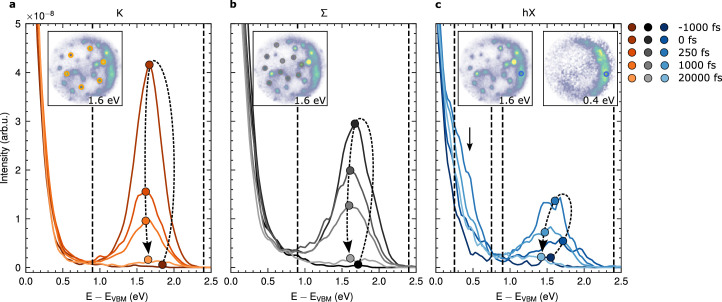
Momentum-filtered energy-distribution-curves of the K-exciton (a), the *Σ* -exciton (b), and the hX (c). The dashed vertical lines indicate the energy range used to extract the delay-dependent data shown in Fig. 5. **c** The vertical black arrow highlights the appearance of the lower energy photoemission maximum that is attributed to the hX photoemission signature.

First, we focus on the WSe_2_ intralayer excitons. Momentum-sharp photoemission features are detected at the K and Σ valleys of the WSe_2_ Brillouin zone (BZ) after optical excitation (Fig. 2a, corners of the orange hexagon and grey circles, respectively; Σ is also labelled as Q or Λ in literature). The K valley spectral weight can be attributed to photoemitted electrons originating from optically bright K-excitons that we excite with the laser pulses (A1s-excitons) and momentum-indirect excitons where the electron and hole components reside at the K and K′ valley, respectively. As photoemission spectral weight from these excitons both appear at the K (K′) valley^30^ and cannot be differentiated within the energy resolution of our experiment^23,31^, we label those jointly as K-excitons (Fig. 2c). Likewise, the Σ valley spectral weight is indicative of the formation of momentum-indirect Σ-excitons whose electron and hole components reside in the Σ and K valley, respectively^22,23,32^. The energetic alignment of the K- and Σ-excitons can be analysed by evaluating momentum-filtered EDCs (Fig. 2e; see Extended Data Fig. 4 for chosen region of interests) and considering the conservation of energy as the EUV laser pulses fragment the excitons into their single-particle electron and hole components in the photoemission process^33^ (${{{E}}}_{{\rm{exc}}}^{{{\rm{K}}}}=1.61\pm 0.05\,\rm{eV}$, ${{{E}}}_{{\rm{exc}}}^{\Sigma }=1.61\pm 0.05\,\rm{eV}$; Extended Data Table 1).

Next, we turn our attention to the two semi-circular photoemission signatures at a radius of $\sqrt{{k}_{x}^{2}+{k}_{y}^{2}}$ ≈ 1.7 Å^−1^ found in momentum maps with centre energies of *E* − *E*_VBM_ = 1.57 ± 0.05 eV (Fig. 2a) and *E* − *E*_VBM_ = 0.38 ± 0.05 eV (Fig. 2b; see corresponding EDCs in Fig. 2e). Such circular structures of spectral weight are characteristic for photoelectrons emitted from molecular orbitals^26,34,35^ (see simulated momentum map in Fig. 2d). Notably, the features are not found in the case of pristine monolayer WSe_2_, but only in the case of the WSe_2_/PTCDA heterostructure (Extended Data Fig. 2 and refs. ^22,23^). Because the excitation energy of 1.7 eV is well below the direct HOMO → lowest unoccupied molecular orbital (LUMO) excitation (≿2 eV; Supplementary Fig. 1 and ref. ^25^), these PTCDA orbital-like photoemission signatures are expected to result from a charge- or energy-transfer process across the TMD/OSC interface and, in consequence, are of major interest to our study.

## A hybrid exciton bridging the 2D–organic interface

**Fig. 3:**
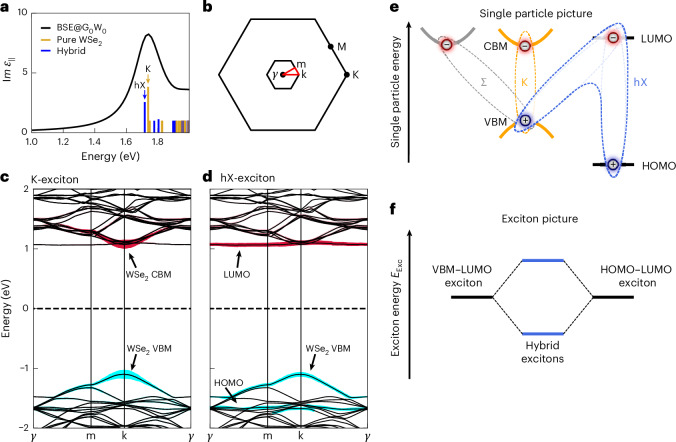
Reciprocal-space representation of the Bloch states and molecular orbitals contributing to the K-exciton and the hX wavefunction. **a**, Absorption spectrum of WSe_2_/PTCDA retrieved by G_0_W_0_ + BSE calculations. The oscillator strengths of the contributing excitons are indicated as solid lines where all values below one (dark-excitons) are set to one for visibility. Excitons with and without contributions from PTCDA orbitals are distinguished in blue and yellow, respectively. **b**, Backfolded BZ according to the theoretical superstructure (Extended Data Fig. 5a). **c**,**d**, The two lowest-lying excitons marked by arrows in **a** are analysed in detail in reciprocal space using the backfolded BZ in **b**. The electron and hole contributions are marked in red and cyan, respectively. While the K-exciton wavefunction is purely composed of TMD valence and conduction band states (WSe_2_ VBM and CBM) (**c**), the hX wavefunction has contributions from the TMD valence bands (WSe_2_ VBM) and from the PTCDA HOMO and LUMO orbitals (**d**). **e**, Visualization of the electron–hole transitions that contribute to the wavefunction of K-exciton, Σ-exciton and hybrid exciton (hX). The hX wavefunction is of partial intra- and interlayer composition and built up by HOMO → LUMO and VBM → LUMO transitions, respectively. **f**, An illustration of the hX in the exciton picture. The intralayer and interlayer electron–hole transitions are expected to be nearly degenerate in energy because of the stronger electron–hole interaction of the pure HOMO–LUMO exciton compared with the VBM–LUMO exciton. Mixing of the transitions leads to the formation of a new bound hybrid excitonic state at lower exciton energies, that is, the hX.

**Extended Data Fig. 5:**
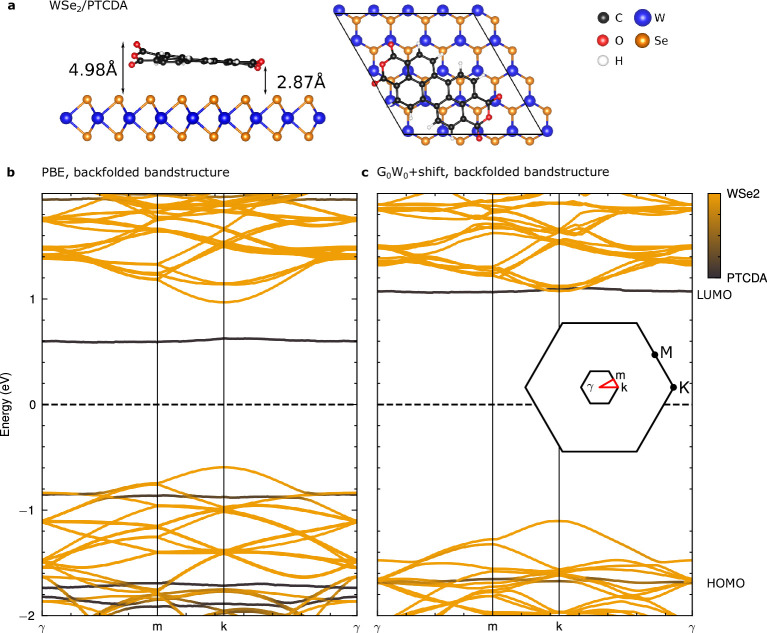
Calculated supercell and single-particle band structure. **a** Optimized structure of PTCDA adsorbed on WSe_2_ monolayer in a 4 × 4 × 1 WSe_2_ supercell. **b**,**c** Band structure of WSe_2_/PTCDA obtained by PBE and G_0_W_0_ calculation analyzed along the directions of the backfolded Brillouin zone indicated in the inset of **c**. The bands are colored according to their PTCDA (black) or WSe_2_ (orange) character. The G_0_W_0_ results in **c** already include the scissors shift applied to the LUMO according to existing STS data in literature^27,28^.

The question at hand is in how far these two fingerprints of molecular orbitals are an indication for multiple excitons with either interlayer or pure PTCDA character, or if they are a fingerprint of so far only predicted hybrid Wannier–Frenkel excitons^3^ with multiple hole contributions^36–39^ from WSe_2_ and PTCDA. To address this question, we start by solving the BSE on top of our *G*_0_*W*_0_ calculations of the WSe_2_/PTCDA heterostructure. For computational feasibility, we neglect spin–orbit coupling (SOC) and adopt a simplified geometry consisting of a single PTCDA molecule in a 4 × 4 × 1 supercell, rather than the experimentally observed herringbone structure (Extended Data Fig. 5a; for a detailed discussion, see the Methods and Supplementary Information). These calculations yield the imaginary part of the in-plane frequency-dependent dielectric function ${\rm{lm}}({\epsilon }_{| | }\left(q\right))$, containing information on optical excitations at the 2D–organic interface, and allows us to identify the band/orbital contributions to the exciton wavefunctions in momentum and real space^15^. In the momentum-direct part of the spectrum (that is, with *q* = *k*_e_ − *k*_h_ = 0), the two lowest-energy excitons are of WSe_2_ (orange, ${E}_{{\rm{exc}}}^{{\rm{K,BSE}}}=1.74\,\rm{eV}$) and hybrid (blue, ${E}_{{\rm{exc}}}^{{\rm{hX,BSE}}}=1.72\,\rm{eV}$) character, respectively (Fig. 3a, marked with arrows). In Fig. 3c,d, the electron and hole contributions to these excitons are analysed in reciprocal space. We find that the 1.74 eV K-exciton is of full WSe_2_ intralayer character and derives its wavefunction purely from WSe_2_ conduction and valence band Bloch states (Fig. 3c). By contrast, for the 1.72 eV exciton, which we term hybrid exciton (hX), the electron component derives its wavefunction from the LUMO of PTCDA, while the hole component has contributions from the WSe_2_ valence bands and also from the PTCDA HOMO (Fig. 3d). In contrast to typical charge-transfer excitons that are exclusively of interlayer type, our G_0_W_0_ + BSE calculations show that the hX is composed of a coherent superposition of intralayer (HOMO → LUMO) and interlayer (VBM → LUMO) contributions. We note that, at first glance, such a mixing might seem counter-intuitive because of the large energy difference of the intra- and interlayer single-particle band gaps (that is, *E*_LUMO_ − *E*_HOMO_ > *E*_LUMO_ − *E*_VBM_; Fig. 3e). However, due to the stronger electron–hole interaction for the case of intralayer HOMO → LUMO transitions as compared with interlayer VBM → LUMO transitions, the individual exciton energies of both electron–hole transitions can be sufficiently degenerate to allow the mixing of both OSC and TMD components (Fig. 3f). Notably, this implies that the hX originates from exciton-level hybridization between interlayer and intralayer excitons, which is possible despite minimal single-particle band hybridization, as demonstrated here.

## Experimental characterization of the hX

**Extended Data Fig. 6:**
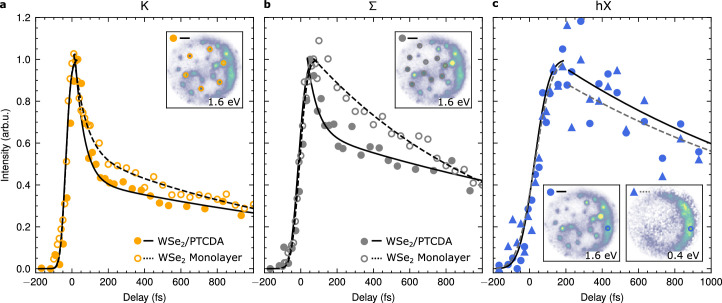
Exciton formation and relaxation dynamics of the bright K-exciton (a), the momentum dark *Σ* -exciton (b) and the hX (c). The respective regions of interest in momentum space are shown in the insets. The time-dependent data collected on WSe_2_/PTCDA (filled symbols) is directly plotted next to data collected on pristine monolayer WSe_2_ (open symbols; data taken from ref. ^23^ The hX double-peak photoemission structure is evaluated separately in energy ranges of 0.9-2.4 eV (circle) and 0.3-0.7 eV (triangles). The rise and decay dynamics are fitted with an error and a (bi-) exponential function, respectively (cf. methods). The fit parameters are summarized in Extended Data Table 1.

The *G*_0_*W*_0_ + BSE prediction of the dual-component character of the hX is in excellent agreement with our experimental findings, as can be verified by analysing three characteristic photoemission fingerprints in the (1) momentum domain, (2) energy domain and (3) time-delay domain. First, the measured momentum maps shown in Fig. 2 are both in agreement with the LUMO orbital momentum map calculated within the framework of photoemission orbital tomography^34^ (Fig. 2d and Extended Data Fig. 2), clearly confirming experimentally that the exciton’s electron component resides in the molecular layer^37^. Second, it is known that photoelectrons that are emitted from excitons are detected one exciton energy ${{{E}}}_{{\rm{exc}}}^{i}$ above the energy of the single-particle bands where the hole component remains after photo excitation^36–38^. Here, we understand the exciton energy ${{{E}}}_{{\rm{exc}}}^{i}$ as the two-particle energy of the electron–hole pair. Thus, for the hX, which has two hole contributions from the WSe_2_ VBM and the PTCDA HOMO (Fig. 3e), one can expect to observe not only one, but two photoemission orbital signatures that are separated in energy by the energy difference between the WSe_2_ VBM and the PTCDA HOMO level: Δ*E*_VBM,HOMO_ = *E*_VBM_ − *E*_HOMO_ = 1.2 ± 0.1 eV. Strikingly, the experimentally found peak-to-peak energy difference Δ*E*_hX_ = 1.18 ± 0.08 eV (Fig. 2e) of the two excitonic photoemission signatures is in quantitative agreement with experimentally retrieved Δ*E*_VBM,HOMO_. Third, if both photoemission signatures result from the break-up of the same hybrid exciton, their population dynamics have to coincide. Indeed, as we will discuss later in detail, the delayed onset and decay dynamics of both photoemission signatures are in quantitative agreement (Extended Data Fig. 6c and Extended Data Table 1). We therefore conclude from experiment and theory that the WSe_2_/PTCDA heterostructure hosts a hybrid exciton whose wavefunction extends across the TMD/OSC interface.

## Real-space wavefunction distribution of hybrid Wannier–Frenkel excitons

**Fig. 4:**
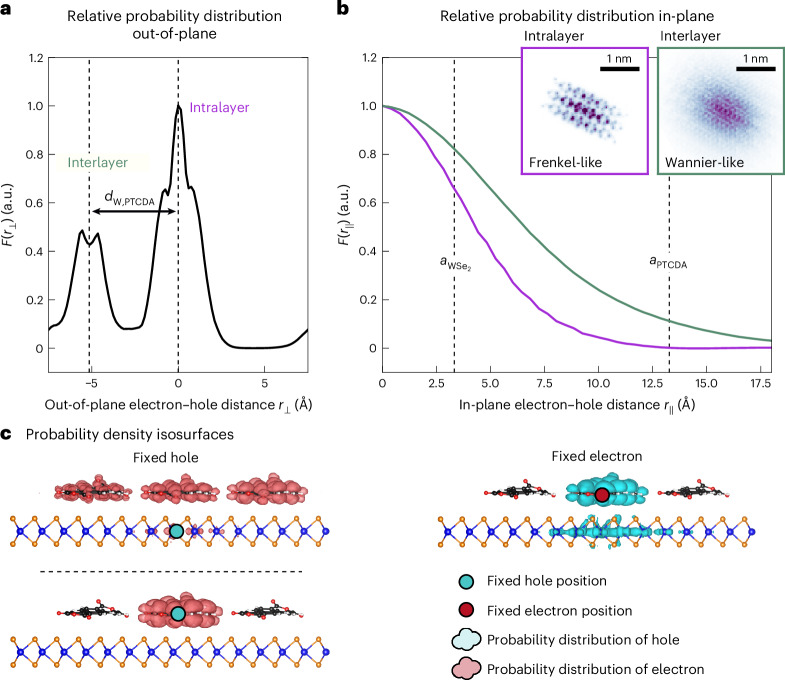
Real-space properties of the hX wavefunction. **a**, The out-of-plane component of the electron–hole correlation function *F*^*i*^(**r**) = *F*(**r**_e_ − **r**_h_) shows two peaks separated by the distance between the tungsten plane and the PTCDA molecule (*d*_W,PTCDA_), which confirms a combination of both intralayer (*r*_⊥_ ≈ 0 Å) and interlayer (*r*_⊥_ ≈ −5 Å) character. **b**, By splitting the in-plane electron–hole correlation into intralayer (purple) and interlayer (green) contributions, it is possible to visualize the major difference in spatial extent. To compare these spatial distributions with the underlying atomic structure, the lattice constants of WSe_2_ and the PTCDA superstructure are indicated as dashed vertical lines. The insets show the *r*_∥_ = (*r*_*x*_,*r*_*y*_) resolved representation of the Wannier-type (green axis) and the Frenkel-type (purple axis) contributions. **c**, Orthographic side view of exemplary probability density isosurfaces for the hX for fixed hole (left) and fixed electron (right) position. Due to the dual Wannier–Frenkel character, the hX probability density isosurfaces depend strongly on the chosen hole location, where the isosurface extends over multiple molecules when the hole is placed in a TMD Bloch state (top), while the isosurface shows clear Frenkel nature when the hole is placed in the PTCDA HOMO (bottom). If the electron is fixed at the molecule (right), the isosurface of the hole has contributions in both the TMD and the PTCDA molecule.

**Extended Data Fig. 7:**
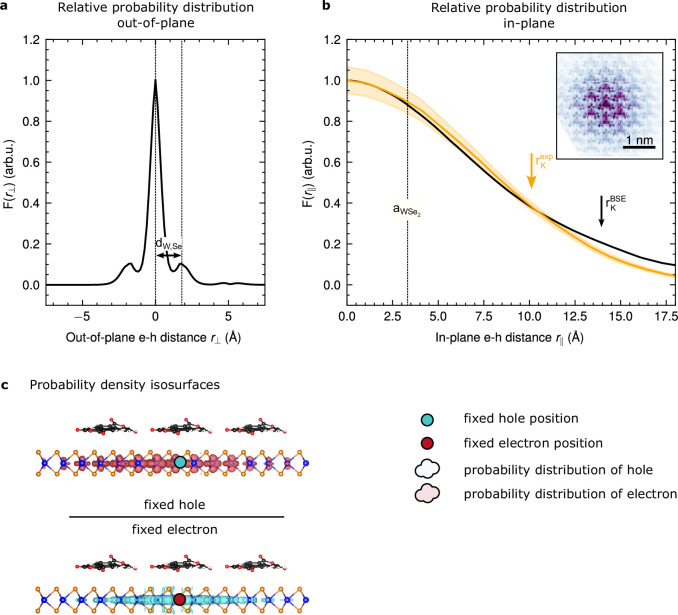
Analysis of the real-space properties of the K-exciton. The relative out-of-plane (**a**) and in-plane components (**b**) of the electron-hole correlation function F^i^(**r**) = F(**r**_*e*_ − **r**_*h*_) are analyzed. a The out-of-plane (*r*_⊥_) component directly confirms the intralayer nature (*r*_⊥_ ≈ 0 Å). Next to the dominant peak at *r*_⊥_ = 0 Å, two side peaks are present at a distance that matches the distance between the tungsten and selenium planes (d_W,Se_) of the WSe_2_ monolayer. **b** The in-plane component can be accessed by theory (black) and experiment (orange). Here, the experimental distribution corresponds to the weighted mean from the angular-averaged probability distribution of all 6 K-points. The shaded area corresponds the standard deviation of the weighted mean of all 6 K-points. The extracted RMS radius is marked by arrows. The inset corresponds to the two-dimensional representation of *F*^K^ (*r*_∥_). **c** Orthographic side view of exemplary probability density isosurfaces of the K-exciton for fixed hole (top) and fixed electron (bottom) position confirming the pure intralayer and Wannier-like character of the K-exciton.

Having access to the excitonic wavefunction contributions of the WSe_2_ Bloch states and the PTCDA orbitals from experiment and theory, we are in the position to evaluate the real-space Frenkel and/or Wannier character of the hX. Specifically, we aim to characterize the exciton’s relative electron–hole distance parallel and perpendicular to the heterostructure in comparison with the size of the WSe_2_ and PTCDA unit cells. Therefore, we analyse the electron–hole correlation function, that is, the probability distribution of the electron–hole separation, *F*^*i*^(**r**) = *F*(**r**_e_ − **r**_h_) with regard to the heterostructure’s out-of-plane (*r*_⊥_; Fig. 4a) and in-plane (*r*_∥_; Fig. 4b) coordinates^40^, which correspond to the exciton’s intra- or interlayer and Frenkel or Wannier character, respectively (see details in the Methods and the analysis for K-exciton in Extended Data Fig. 7).

Intriguingly, for the out-of-plane component (Fig. 4a), there is not only a peak around *r*_⊥_ ≈ 0 Å that indicates intralayer character, but also a peak centred at *r*_⊥_ ≈ −5 Å, which matches the distance between the tungsten plane and the PTCDA molecule and therefore indicates the additional interlayer character of the hX. Hence, the double-peak structure in Fig. 4a is a direct signature of the mixed intra- and interlayer contributions to the hX.

**Extended Data Fig. 8:**
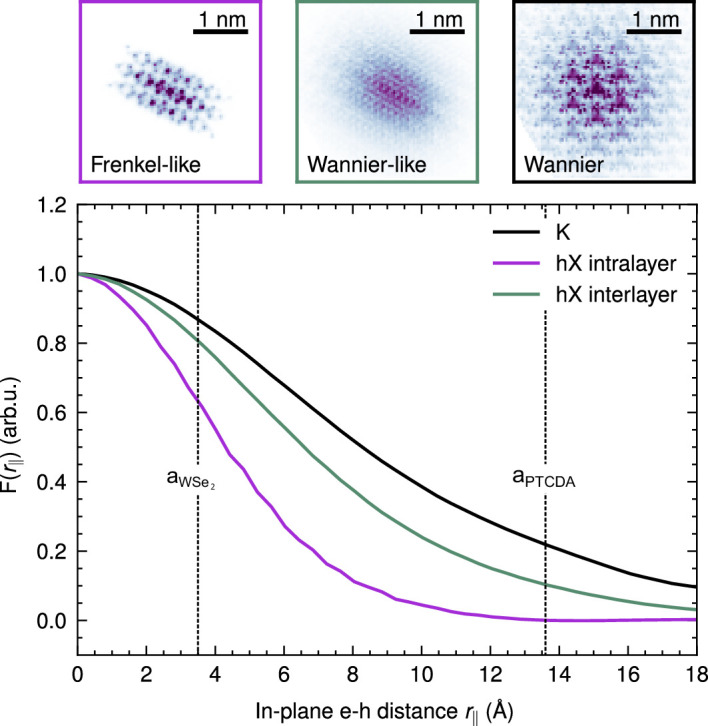
Comparison of the in-plane correlation function of the K exciton, the intralayer and interlayer component of the hX. For better comparison, each component is normalized individually. The upper row (from left to right) shows the two-dimensional in-plane correlation function of the intra- and interlayer components of the hX and of the K-exciton. The line profiles correspond to the respective angular-averaged Fourier filtered in-plane correlation function.

Complementary, the in-plane electron–hole distribution function *F*^*i*^(*r*_∥_) (Fig. 4b) contains information on the Frenkel and/or Wannier character of the hX. Here, we plot the intra- and interlayer contributions separately as purple and green lines in Fig. 4b. The relative probability of the intralayer contribution is almost entirely (to 99%) confined to values smaller than the lattice constant of the PTCDA supercell (*a*_PTCDA_), indicating a Frenkel-like character. By contrast, only 82% of the interlayer contribution is confined to values smaller than *a*_PTCDA_. Hence, similarly to the Wannier K-exciton, the interlayer component of the hX extends over multiple PTCDA unit cells (see Extended Data Table 2 and Extended Data Fig. 8 for a direct comparison), thereby exhibiting a more Wannier-like character. The different character is even more evident when considering the 2D representations of *F*^hX^(*r*_∥_) in the insets in Fig. 4b, which show that the interlayer component resembles the overall Gaussian intensity distribution known for a K-exciton (Extended Data Fig. 7 and refs. ^31,41,42^), while the Frenkel component shows a much stronger spatial structuring that stems from the molecular orbital.

To further illustrate the spatial extent of the hX—both in terms of intra- and interlayer contributions, as well as its Wannier and Frenkel character—Fig. 4c shows exemplary probability density isosurfaces of the exciton’s electron and hole components (red-shaded and cyan-shaded volumes) that are obtained by fixing the exciton’s hole (cyan dots) or electron components (red dot) at typical positions in the heterostructure. When the hole is placed in a delocalized state in the TMD layer (top left), we find a Wannier-like isosurface where the electron is spread over multiple PTCDA molecules. By contrast, when the hole is placed on the PTCDA molecule (bottom left), the electron is completely localized on the same molecule, too. In other words, the isosurface now describes a Frenkel-type exciton. Most interestingly, when the electron is fixed on the PTCDA layer (right), the hole isosurface again shows the dual component characteristics with a localized part on the same PTCDA molecule, and also a (weaker) delocalized contribution on the TMD layer. Hence, the hole isosurface has both Wannier and Frenkel character. This hybrid intra- and interlayer and Frenkel and Wannier nature of the hX is a unique feature of the TMD/OSC interface that highlights the versatility of these combined platforms for controlling optoelectronic energy conversion pathways.

## Femtosecond time- and orbital-resolved exciton dynamics

**Fig. 5:**
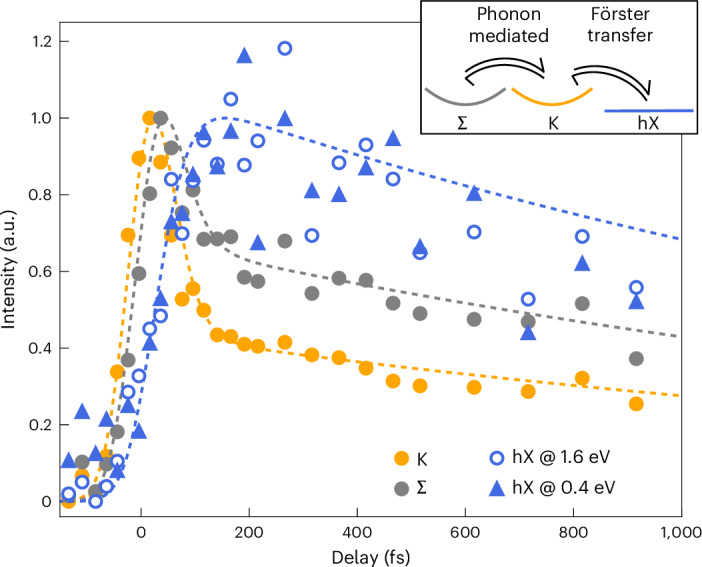
Femtosecond formation of the hX at the hybrid WSe_2_/PTCDA interface. Subsequent to the optical excitation of WSe_2_ K-excitons (orange), exciton–phonon scattering and Förster-type dipole–dipole interactions lead to the formation of Σ-excitons (grey) and the hX (blue), respectively. The open circles and filled triangles label the photoemission intensity obtained from the upper and the lower energy photoemission peak from the hX (Fig. 2e), respectively. The symbols encode photoemission spectral weight filtered in energy and momentum space (Extended Data Fig. 4), and the dashed lines are guides to the eye. A steady state between all excitonic occupations is reached after 150 fs (inset), which decays on the 2-ps timescale. The formation and decay rates of all excitonic photoemission signatures are quantified in Extended Data Fig. 6 and summarized in Extended Data Table 1.

Finally, we want to elucidate the ultrafast formation and thermalization dynamics of all excitons involved. Figure 5 shows the femtosecond pump–probe delay dependence of photoemission spectral weight from K-excitons (K valley), Σ-excitons (Σ valley) and hXs (molecular features hX@1.6 eV and hX@0.4 eV). Subsequent to the optical excitation of K-excitons and the concomitant rise of spectral weight at the K valley (orange), the photoemission intensities at the Σ valley (grey) and for the molecular features (blue) rise with delayed onsets of 22 ± 4 fs and 61 ± 10 fs, respectively. The molecular features’ spectral weight peaks at about 150 fs, and their decay can be well described with a single-exponential function with a decay constant of ${\tau }_{{\rm{decay}}}^{{\rm{hX}}}=1.8\pm 0.7\,\rm{ps}$. Interestingly, following the increase of the hX spectral weight, the initially fast decay of the K and Σ valley spectral weight is slowed down and shows the same behaviour as the decay of the hX on longer timescales. This is confirmed by fitting bi-exponential functions to the decay of the K- and the Σ-exciton, which show slow decay constants of ${\tau }_{{\rm{slow}}}^{{\rm{K}}}=2.1\pm 0.4\,\rm{ps}$ and ${\tau }_{{\rm{slow}}}^{{\rm{\Sigma }}}=2.2\pm 0.4\,\rm{ps}$, respectively (Extended Data Table 1). From this analysis, we conclude that subsequent to the optical excitation of K-excitons and on a sub-200-fs timescale, a Σ *⇌* K *⇌* hX steady-state population with common decay channels is established.

However, while in pure TMD heterostructures charge and energy transfer across interfaces is mediated and explained by band structure hybridization effects^31,43–45^, the general preconception for TMD/OSC interfaces is that orbital hybridization is weak^4,46^ and that, as a consequence, related charge-transfer processes or Dexter-type energy-transfer processes that require wavefunction overlap^47^ are less likely. Indeed, neither experimental nor theoretical investigations reveal signatures of substantial orbital hybridization at the TMD/OSC interface: the experimentally determined K- and Σ-exciton energies are in quantitative agreement with ARPES^23^ and photoluminescence^48^ data reported for monolayer WSe_2_ (Extended Data Table 1), that is, without a molecular overlayer. Specifically, compared with ${{{E}}}_{{\rm{exc}}}^{\rm{K}}$, no reduction of ${{{E}}}_{{\rm{exc}}}^{\Sigma }$ due to the PTCDA adsorption is observed, as is the case of pure TMD few-layer systems with hybridized bands^23^. Hence, the experimental results imply that the K- and Σ-exciton wavefunctions are of mere WSe_2_ intralayer character, with negligible contribution from PTCDA orbitals. This is in agreement with our density functional theory (DFT) calculations that also do not show any hybridization of the single particle PTCDA LUMO and WSe_2_ conduction band states (Extended Data Fig. 5c). In consequence, the formation of the hX cannot be mediated by hybridized states between the layers. Instead, we conclude that energy transfer must be dominantly mediated by dipole–dipole interactions, that is, in a Förster-type energy transfer process. Indeed, all requirements for such a process are fulfilled^8,47^: First, the K and hX energies are close in energy, satisfying the requirement of energy conservation (Fig. 2c and Extended Data Table 1). Second, the dipole moment of the HOMO–LUMO contribution to the hX is polarized in-plane, facilitating the coupling to the in-plane dipole moment of the WSe_2_ K-exciton. Third, because of the mixed nature of the hX wavefunction and the contribution of HOMO–LUMO transitions, our *G*_0_*W*_0_ + BSE calculations predict that the oscillator strength is rather large (Fig. 3a), making the Förster-type process efficient. We note, however, that the hX formation is one to two orders of magnitude faster than typically found in other reports discussing the dynamics of Förster processes at TMD/OSC^47^ or TMD/graphene^49,50^ interfaces. We suspect that the fast rise time of the hX might be related to the fact that the dipole–dipole interactions do not induce a complete energy transfer across the 2D–organic interface. Instead, the dipole–dipole interactions promote the conversion of WSe_2_ intralayer K-excitons to hXs, whose wavefunction is composed of PTCDA intralayer HOMO/LUMO and interlayer WSe_2_-VBM/LUMO orbital contributions. While future theoretical work is needed to verify the timescale of the Förster-type energy-transfer process at the WSe_2_/PTCDA interface, our work already highlights how excitonic wavefunction engineering can directly contribute to efficient energy transfer processes in 2D–organic hybrid heterostructures.

## Methods

### Sample preparation

To fabricate the WSe_2_/PTCDA heterostructure (Extended Data Fig. 1a), hBN was first exfoliated onto a 0.1% niobium-doped SrTiO_3_ substrate and an approximately 50-nm-thick flake was identified by optical microscopy. In a parallel procedure, WSe_2_ monolayers were directly exfoliated onto a silicone gel film (DGL Film, Gel-Pak) and identified through optical microscopy and Raman spectroscopy. Afterwards, a monolayer WSe_2_ flake was transferred from the silicone gel film onto the hBN flake on the SrTiO_3_ substrate (Extended Data Fig. 1b). After introduction into ultrahigh vacuum (<5 × 10^−9^ mbar), the sample was annealed at 670 K for 2 h to ensure a clean sample surface. The bare monolayer WSe_2_ was analysed with the momentum microscope in real space (Extended Data Fig. 1c) and reciprocal space (Extended Data Fig. 2e), showing the expected characteristic features of monolayer WSe_2_, that is, the spin-split valence bands at the K valley and a single parabolic band at the Γ valley below the global VBM at the K valley (Fig. 1b and refs. ^20,23^). Moreover, the clear separation between the top and the bottom valence band (Fig. 1c) indicates the high-quality of the WSe_2_ with only contributions of inhomogeneous broadening.

Subsequently, approximately a monolayer of PTCDA was thermally evaporated onto the sample, which was maintained at room temperature (base pressure <1 × 10^−9^ mbar). The deposition rate was monitored with a with a quartz crystal microbalance and calibrated using the known deposition of PTCDA onto a Ag(110) crystal surface. On the Ag(110) surface, the first monolayer of PTCDA is adsorbed in a brickwall structure whereas additional layers grow in a Herringbone structure with a different superstructure that can be analysed by low energy electron diffraction (LEED)^51^. By step-wise evaporation of PTCDA onto Ag(110) and recording of the LEED pattern, the evaporation rate was determined and was then used to deposit a monolayer PTCDA onto monolayer WSe_2_. The successful deposition of a monolayer PTCDA onto monolayer WSe_2_ was confirmed by the observation of additional spectral weight in the static ARPES data, which can be attributed to the HOMO of PTCDA (Fig. 1c,d) and backfolded WSe_2_ bands (Extended Data Fig. 1e), which are caused by the adsorbed molecular PTCDA superstructure (Fig. 1b). The superstructure matrix

$$M=\left[\begin{array}{cc}1.58&6.78\\ 4.39&1.32\end{array}\right]$$

was determined by LEED on a cleaved WSe_2_ bulk crystal (Extended Data Fig. 1d) and is in good agreement with ref. ^28^ and the ARPES data (Extended Data Fig. 1e).

### Femtosecond momentum microscopy

All photoemission data were acquired with the Göttingen in-house photoemission set-up^17,52^ that combines a time-of-flight momentum microscope^53^ (Surface Concept GmBH, ToF-MM) with a 500-kHz high-harmonic generation beamline (26.5 eV *p*-polarized, 20 fs). For the time-resolved measurements, the photon energy of the *s*-polarized pump was tuned to *hν* = 1.7 eV with 50 ± 5 fs pulse duration using an optical parametric amplifier. The experimental set-up is described in detail in ref. ^17^. The pump fluence was adjusted to 280 ± 20 μJ cm^−2^, which results approximately in an initial K-exciton density of (5.4 ± 1.0) × 10^12^ cm^−2^ (refs. ^31,48^). All experiments are performed with an energy, momentum and time resolution of 200 ± 30 meV, 0.04 ± 0.01 Å^−1^ and 54 ± 7 fs (refs. ^17,31,54^). The static measurements (Fig. 1c) were performed at *T* = 50 K, while all pump–probe delay-dependent measurements were performed at room temperature (300 K).

### Photoemission data processing

The time-of-flight momentum microscope enables the simultaneous measurement of the kinetic energy and both in-plane momenta of the photoemitted electrons^53^. However, the acquired three-dimensional photoemission data are affected by various lens aberrations and other distortions, such as pump- and probe-induced space-charge effects and surface photovoltage^55–57^. Therefore, the photoemission data needs to be preprocessed before further evaluation by (1) correcting a time-dependent rigid energy shift and (2) correcting for distortions that are induced by the projection and focal lens system.

First, the time-dependent energy shift was corrected by minimizing the variance between the momentum-integrated spectra for *E* − *E*_VBM_ < 1.8 eV. Second, an additional measurement was performed with a grid inserted in the Fourier plane^58,59^. We then determined the parameters for an affine transformation that maps the measured data onto an undistorted and energy-independent grid. This transformation is applied to all datasets. Small remaining distortions induced by the first lens system were corrected by fitting the positions of the K-excitons and mapping them onto an equilateral hexagon. The same positions were used to perform the momentum calibration using the lattice constant of ${{\rm{WSe}}}_{2} \, {a}_{{{\rm{WSe}}}_{2}}=0.3297\,\rm{nm}$. In addition, for each delay step, the data were momentum-wise normalized to the energy range between *E* − *E*_VBM_ = −1.8 and −3.8 eV. This momentum-wise normalization accounts for potential changes in illumination due to possible instabilities during the long integration times of the time-resolved measurements.

### Quantitative analysis of the exciton energies and dynamics

The EUV laser pulses fragment the Coulomb-bound electron–hole pairs into their single-particle components. As this process conserves energy and momentum^33,60,61^, the exciton energies *E*_exc_ can be extracted by fitting the delay-integrated (100–500 fs), background-substracted (see the non-negative matrix formalism (NMF) method below) and momentum-filtered EDCs shown in Fig. 2c with either one (K- and Σ-excitons) or two (hX) Gaussian peaks *I*_p_ and an exponential background *I*_bg_, that is,

1 $${I}_{{\rm{p}}}(E\;)=\frac{A}{\sigma \sqrt{2\uppi }}\exp \left\{\left(-\frac{{(E-\mu )}^{2}}{2{\sigma }^{2}}\right)\right\},$$

2 $${I}_{{\rm{bg}}}(E\;)={A}_{{\rm{bg}}}\exp \left\{\left(-\frac{E}{{\tau }_{{\rm{bg}}}}\right)\right\}.$$

The extracted peak energies *E* − *E*_VBM_ of the K and *Σ* excitons directly correspond to the exciton energies *E*_exc_ because the hole resides at the VBM of WSe_2_. For the hX, the peak energy of the higher lying peak at *E* − *E*_VBM_ = 1.57 ± 0.05 eV directly corresponds to ${{{E}}}_{{\rm{exc}}}^{{\rm{hX}}}$, whereas the lower-energy peak at *E* − *E*_VBM_ = 0.38 ± 0.05 eV has to be referenced to the HOMO at *E* − *E*_VBM_ = −1.2 ± 0.1 eV, which results in the same exciton energy ${{{E}}}_{{\rm{exc}}}^{{\rm{hX}}}$ = 1.58 ± 0.1 eV. In Extended Data Table 1, the quantified exciton energies ${{{E}}}_{{\rm{exc}}}^{i}$ are compared with the BSE@*G*_0_*W*_0_ calculations and ARPES and photoluminesence experiments on monolayer WSe_2_ (refs. ^22,23,62^). The total error of the experimental values is estimated to be approximately 0.05 eV, taking into account fitting errors and possible errors induced by the energy calibration and space charge effects.

To analyse the exciton dynamics of the K, Σ and hX photoemission signatures (Fig. 5), we filter the raw photoemission data, that is, without background subtraction, by their energy and momentum coordinate. The respective EDCs and the chosen region of interests are shown in Extended Data Fig. 4. To quantify the rise time, we fitted the energy- and momentum-filtered time-resolved photoemission spectral weight traces with an error function

3 $$I(t)=\frac{1}{2}\left(1+\,\text{erf}\,\left(\frac{t-{\mu }_{{\rm{onset}}}}{\sqrt{2}{\sigma }_{{\rm{rise}}}}\right)\right)$$

in the delay regions −200 fs to 0 fs, −200 fs to 20 fs and −200 fs to 150 fs, respectively (Extended Data Fig. 6). Here, *μ*_onset_ indicates the onset time, while *σ*_rise_ is directly related to the rise time.

Similarly, we fitted the decay of photoemission spectral weight with a bi-exponential decay (equation (4)) between 0 fs and 2,000 fs and between 20 fs and 2,000 fs for the K- and Σ-excitons, respectively. The hX was fitted with a single exponential decay function (equation (5)) between 200 fs and 2,000 fs:

4 $$I(t)=A\left(\frac{1}{1+f}\exp \left\{\left(-\frac{t}{{\tau }_{{\rm{fast}}}}\right)\right\}+\frac{f}{1+f}\left(-\frac{x}{{\tau }_{{\rm{slow}}}}\right)\right),$$

5 $$I(t)=A\exp \left\{\left(-\frac{t}{{\tau }_{{\rm{slow}}}}\right)\right\}.$$

The relevant time constants are given in Extended Data Table 1.

### Non-negative matrix factorization

Due to the small size of the WSe_2_ monolayer and the small real-space selection aperture (10 μm effective diameter), the measurement was susceptible to a time-independent background intensity. Therefore, the excited state momentum maps and momentum-filtered EDCs shown in Fig. 2 and in the insets of Extended Data Fig. 4 and 6 were background subtracted.

For the background determination, we used NMF, as implemented in the scikit-learn package for Python^63^. NMF is a dimensionality-reduction method that so far remains unexplored in time-resolved ARPES, but it has recently found application in spatially resolved material science studies based on X-ray diffraction^64^ and also static ARPES experiments^65^. Similar to principal component analysis, NMF is based on the numerical factorization of a given matrix *X* into two matrices *W* and *H*, with the additional condition that all matrices have only non-negative elements. In our case, *X* is given by the time-dependent raw dataset where we consider only excited state data above *E* − *E*_VBM_ = 0.15 eV. In addition, we fix *W* by a static background and the four extracted time traces of the K, Σ, *hX@*1.6 eV and *hX@*0.4 eV photoemission signal plotted in Fig. 5. The determined output then is the matrix *H* that consists of five components, each following one of the four given time dependencies and the time-independent background. Extended Data Fig. 3 shows extracted components integrated over the regions of interest in energy. Notably, the different components 1–4 can be assigned in reasonable agreement to the different excitonic photoemission signatures despite the strong overlap in time, energy and momentum (orange hexagons, grey circles and blue circle). Component 5 is time-independent and used for background substraction.

### Photoemission orbital tomography

Using the plane-wave model of photoemission, the measured momentum-dependent photoemission intensity of electrons emitted from molecular orbital can be expressed as^34^

6 $$I({\bf{k}})={\left\vert {\bf{A}}\cdot {\bf{k}}\right\vert }^{2}{\left\vert {\mathscr{F}}\left(\psi ({\bf{r}})\right)\right\vert }^{2}\delta \left({E}_{\rm{b}}+{E}_{{\rm{kin}}}+\varPhi -h\nu \right),$$

where *ψ*(**r**) is the real-space electronic wavefunction, ${\mathscr{F}}$ is the Fourier transform and ${\left\vert {\bf{A}}\cdot {\bf{k}}\right\vert }^{2}$ is a polarization factor defined by the vector potential **A** of the incoming electromagnetic field. The Dirac *δ* function ensures energy conservation of the photoemission process, which includes the photon energy *hν*, the electron binding energy *E*_b_, the work function *Φ* and the kinetic energy *E*_kin_ of the emitted photoelectron. This model has been successfully applied to analyse orbital wavefunctions of transient excited states in PTCDA^26^ and extended to the description of the photoemission signature of excitons in C_60_ (ref. ^36^). According to refs. ^36,37^, the photoemission signature of the hX with multiple hole contributions, but only a single electron contribution, must feature a two-peak structure, where the momentum distribution of both peaks resembles the Fourier transform of the LUMO of PTCDA as described by equation (6). Based on this model, we calculate the expected momentum map of the HOMO and the hX considering all the different orientations of the PTCDA molecule^28^. The real-space molecular orbitals calculated by DFT are extracted from ref. ^66^. The results are plotted in Extended Data Fig. 2c,g. We note that the theoretical momentum fingerprints were calculated for single-particle electrons (that is, using the Kohn–Sham orbitals). In the near future, progress in the field of exciton photoemission orbital tomography^36,37^ may enable the calculation of predicted momentum fingerprints also for excitonic states; however, such calculations are currently not possible for the present WSe_2_/PTCDA structure.

### Calculation of the electronic structure

A *G*_0_*W*_0_ treatment of the herringbone-type WSe_2_/PTCDA heterostructure is beyond current computational possibilities. Instead, to meet the experimental conditions as closely as possible, we consider a configuration of a PTCDA molecule adsorbed on a 4 × 4 × 1 supercell of the pristine WSe_2_ structure with an in-plane lattice parameter of 3.317 Å (Extended Data Fig. 5; for a detailed discussion of the implemented supercell, refer to the Supplementary Information). We optimize the atomic structure, consisting of 86 atoms, using the all-electron code ‘FHI-aims’^67^ by minimizing the amplitude of the interatomic forces below a threshold value of 10^−3^ eV Å^−1^. For all species a tight basis is used. The resulting adsorption geometry is shown in Extended Data Fig. 5a. The PTCDA molecule is slightly tilted, with the shortest and longest distance to the substrate being 2.87 Å and 4.98 Å, respectively, measured from the top of the substrate.

The ground-state, *G*_0_*W*_0_ and BSE calculations are performed using the all electron full-potential code ‘exciting’^29^, which implements the family of linearized augmented plane wave plus local orbitals (LAPW+LO) methods. The muffin-tin spheres of the inorganic component are chosen to have equal radii of 2.2 bohr. For PTCDA, the radii are 0.9 bohr for hydrogen (H), 1.1 bohr for carbon (C) and 1.2 for oxygen (O). The electronic properties are calculated first using DFT with the generalized gradient approximation in the Perdew–Burke–Ernzerhof parametrization for the exchange–correlation (xc) functional. The sampling of the BZ is carried out with a homogeneous 3 × 3 × 1 Monkhorst–Pack **k**-point grid. To account for van der Waals forces and intermolecular interactions, we adopt the Tkatchenko–Scheffler method^68^. The quasi-particle (QP) energies are computed within the *G*_0_*W*_0_ approximation^69^, where we include 200 empty states to compute the frequency-dependent dielectric screening within the random-phase approximation. A 2D truncation of the Coulomb potential in the out-of-plane direction *z* is used^70^. The band structure is computed by using interpolation with maximally localized Wannier functions^71^ and Fourier interpolation (Extended Data Fig. 5b and Extended Data Fig. 5c, respectively). To keep the calculations feasible, SOC is not considered in this work. Although SOC leads to a splitting of the lowest-energy excitonic peak by approximately 450 meV (ref. ^72^), it would not alter the type-I level alignment of WSe_2_/PTCDA. Importantly, the molecular states involved in the formation of the hX, that is, the HOMO and LUMO, would not be affected by the inclusion of SOC.

To allow a direct comparison with the experimental ARPES data (Fig. 1d), we unfold the theoretical band structure by symmetry mapping of the Bloch-vector-dependent quantities defined in the supercell into the unit-cell calculations. Here, the wavefunctions are constructed in a uniform real-space grid of 120 × 120 × 120 and used to calculate the spectral function (Fig. 1f).

The QP band gap of WSe_2_ in the heterostructure is in good agreement with that measured by STS^28^; however, the PTCDA gap is underestimated, which is most evident in the level alignment of the HOMO. This discrepancy can be explained by the interplay of different effects such as SOC, the choice of the xc functional and its role as a starting point for the QP calculations, and the interlayer distance between PTCDA and WSe_2_. Uncertainties in the latter can be related to packing density, the xc functional and the treatment of van der Waals interactions, or temperature^73^. In earlier studies on ZnO/WSe_2_ it has been shown that increasing the interlayer distance leads to a noticeable increase in the QP gaps on both sides of the interface^74^. Also in WSe_2_/PTCDA, increasing (decreasing) the interlayer distance will decrease (increase) the mutual screening, leading to an increase (decrease) in the HOMO–LUMO gap. This, in turn, would lead to an increase (decrease) in the VBM–HOMO distance. Overall, there is an interplay of effects on the order of a few tenths of an electronvolt each, which can only be resolved through extensive future QP calculations.

To overcome this mismatch, we apply a scissors shift to the molecular levels. Shifting the LUMO by −50 meV closer to the experimental value^28^ results in very good agreement of the excitonic spectrum with experiment (see below).

### Calculation of the exciton spectrum

For the calculation of the exciton spectrum, we solve the BSE on top of the QP band structure, where the screened Coulomb potential is computed using 100 empty bands. In the construction and diagonalization of the BSE Hamiltonian, 16 occupied and 14 unoccupied bands are included, and a 12 × 12 × 1 shifted **k**-point mesh is adopted. Calculations are performed using the BSE module^75^ of the ‘exciting’ code.

### Calculation of the correlation function

Following the definition in ref. ^40^, we calculate the electron–hole correlation function

7 $${F}^{i}(\bf{r})={\int}_{\varOmega }{d}^{\,3}{{\bf{r}}}_{\rm{e}}| {\psi }_{i}({{\bf{r}}}_{\rm{h}}={{\bf{r}}}_{\rm{e}}+{\bf{r}},{{\bf{r}}}_{\rm{e}}){| }^{2},$$

where *F*^*i*^ describes the probability of finding electron and hole separated by the vector **r** = **r**_h_ − **r**_e_. We approximate this integral by a discrete sum over a finite number of fixed electron coordinates. For each electron position, the hole probability ∣*ψ*_*i*_(**r**_h_ = **r**_e_ + **r**, **r**_e_)∣^2^ is computed on an evenly spaced, dense grid of 100 × 100 × 100 sampling points, covering approximately 3 × 3 × 1 supercells. For the hX, we sampled 60 positions on the PTCDA molecule (0.5 Å^−1^ below and above the carbon and oxygen atoms) because its electronic contribution is almost entirely composed of the LUMO of PTCDA. Similarly, we calculated the electron–hole correlation function of the K-exciton (Extended Data Fig. 7), which is completely localized in the WSe_2_ layer. Here, we sampled 16 positions close to the W atoms where we expect a high probability of finding the electron.

For further analysis, the three-dimensional correlation function is split into its in-plane and out-of-plane components by integrating over the other direction (Fig. 4 and Extended Data Fig. 7). Here, the intralayer (purple) and interlayer (green) in-plane distributions were extracted by integrating exclusively over the respective peak of *F*(*r*_⊥_), that is, *r*_⊥_ = −3 to 4.5 Å and *r*_⊥_ = −10.5 to −3 Å for the intra- and interlayer components, respectively. Notably, the in-plane component shows a distinct periodic pattern (see insets in Fig. 4b and Extended Data Fig. 7b). Thus, to extract the in-plane radial profile and the root mean square (RMS) radius, we first filter the data in Fourier space, thereby smoothing it in the real space.

### Spatial analysis of the K-exciton and comparison with hX

In analogy to the analysis of the spatial structure of the hX in Fig. 4, we analyse the K-exciton wavefunction (Extended Data Fig. 7). From the BSE calculation, we find that *F*^K^(*r*_⊥_) is dominated by a single peaked feature centred around *r*_⊥_ ≈ 0 Å, implying that the exciton is of pure intralayer character (Extended Data Fig. 7a). Consequently, the probability density of finding K-excitons in the WSe_2_ layer is nearly 100%. Consistent with this, the two smaller side peaks located at a distance corresponding to the distance between the tungsten and selenium planes *d*_W,Se_, can be attributed to a residual probability of the electron and/or hole being at the selenium atoms.

Due to its hydrogen-like structure, the in-plane electron–hole probability distribution of the K-exciton can be directly reconstructed from the experimental photoemission momentum fingerprint via Fourier analysis^31,41,42,59^. This allows a direct comparison of *F*^K^(**r**_∥_) between theory and experiment (Extended Data Fig. 7b). Both theory and experiment confirm the pure Wannier-like character of the K-exciton because the radial distribution is much larger than the WSe_2_ lattice constant. For a more quantitative analysis, we compare the extracted RMS radii to be (${{{r}}}_{{\rm{K}}}^{{\rm{BSE}}}$ = 14 Å) (theory) and ${{{r}}}_{{\rm{K}}}^{\exp }$ = 10 ± 1 Å (experiment), which are in excellent agreement. Note that, due to the finite momentum resolution of the photoemission signal, the derived RMS radius represents a lower limit of the true value. The distinct intralayer and Wannier-like character of the K-exciton can be further visualized by plotting the isosurface of a representative fixed electron and hole position (Extended Data Fig. 7c), which stands in clear contrast to the isosurfaces of the hX (Fig. 4).

After having identified the K-exciton as a Wannier exciton, we compare its in-plane correlation function directly to that of the inter- and intralayer contributions to the hX (Extended Data Fig. 8). Here, we find that the interlayer contribution resembles the spatial distribution of the K-exciton, while the intralayer contribution is more localized and exhibits a stronger spatial modulation stemming from the molecular orbitals. This direct comparison confirms the previously assigned Frenkel- or Wannier-type character of the intra- and interlayer contribution of the hX.

For comparison, the calculated RMS radii of the K-exciton and both components of the hX are summarized in Extended Data Table 2.

## Online content

Any methods, additional references, Nature Portfolio reporting summaries, source data, extended data, supplementary information, acknowledgements, peer review information; details of author contributions and competing interests; and statements of data and code availability are available at 10.1038/s41567-025-03075-5.

## Supplementary information

Supplementary Information Supplementary Fig. 1 and Discussion.

## Figures and Tables

**Extended Data Table 1:**
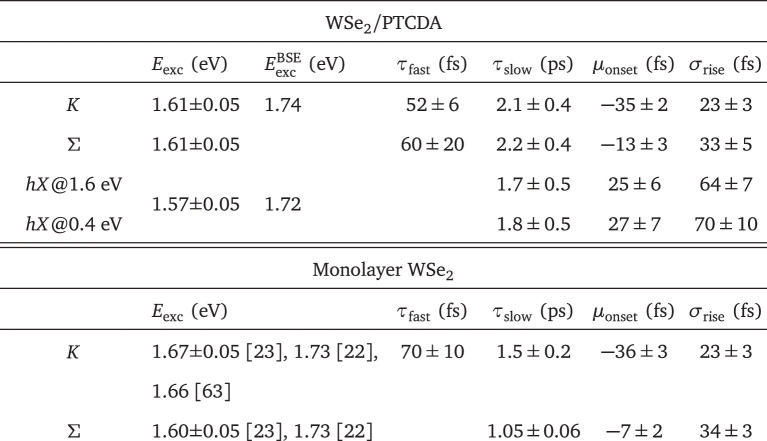
Experimental and theoretical exciton energies (${E}_{exc},{E}_{exc}^{{\rm{BSE}}}$), as well as decay (*τ*_fast_, *τ*_slow_) and rise (*μ*_onset_, *σ*_rise_) times of the excitons found in WSe_2_/PTCDA

The exciton energies are compared to those of pure monolayer WSe_2_ taken from literature as indicated in the table^22,23,62^. The decay- and rise-times are compared to those pure monolayer WSe_2_ determined by the same fitting routine based on data from ref. ^23^. Details of the applied fitting procedures can be found in the Method section.

**Extended Data Table 2:**
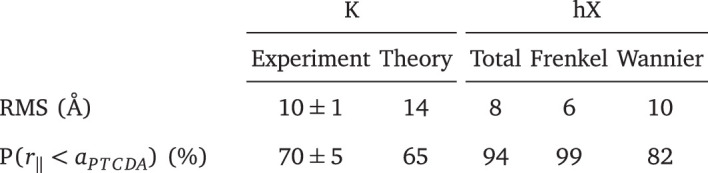
omparison of the spatial extent of the K-exciton and the hX

Theoretical and experimental extracted RMS radii of the in-plane relative probability distribution of the K-exciton and the hX, as well as the probability that the in-plane relative electron-hole distance is smaller than the PTCDA lattice constant (P(*r*_∥_ < *a*_PTCDA_)).

## Data Availability

The datasets that support the experimental findings of this study are available via GRO.data at 10.25625/BEKR3I (ref. ^76^). The theoretical data are available via NOMAD at 10.17172/NOMAD/2024.10.11-1 (ref. ^77^).
