# Supplementary material for: Hybrid Frenkel–Wannier excitons facilitate ultrafast energy transfer at a 2D–organic interface
Source: Nat Phys. 2025 Oct 29;21(12):1973–80. doi: 10.1038/s41567-025-03075-5 (PMC12695656; doi:10.1038/s41567-025-03075-5)
Supplement: Supplementary file 1 — Supplementary Fig. 1 and Discussion. [file 41567_2025_3075_MOESM1_ESM.pdf]

nature physics

Supplementary information

https://doi.org/10.1038/s41567-025-03075-5

Hybrid Frenkel–Wannier excitons facilitate ultrafast energy transfer at a 2D–organic interface

In the format provided by the authors and unedited

Comparison of brick-wall and herringbone geometries of PTCDA

For a feasible computational approach, still capturing all the essential features of the electronic and optical properties, we have chosen a 4×4×1 supercell of WSe_2 with a PTCDA monolayer with one molecule per unit cell adsorbed. To support this choice, we compare this so-called brick-wall structure of PTCDA with the herringbone PTCDA arrangement, which is observed experimentally. Supplementary Fig. 1 (top) shows that the Kohn-Sham band structures are very similar, with direct gaps of 1.46 eV and 1.37 eV, respectively. The optical spectra calculated using BSE are shown in Supplementary Fig. 1 (middle). For the brick-wall geometry, the calculation was carried out on top of the quasi-particle bands obtained by G_0W_0, while for the herringbone arrangement, a scissor shift was used. The imaginary parts of the dielectric function exhibit negligible differences only in peak height, within the energy range between 0 and 4 eV. In both systems, the real space representation of the lowest-energy exciton (Supplementary Fig. 1 (bottom)) manifests Frenkel character, with the electron distribution localized on one molecule. Based on this comparison, we expect that the results for WSe_2/PTCDA in the herringbone arrangement would show the same behavior as that of the brick-wall unit cell discussed in the main text.

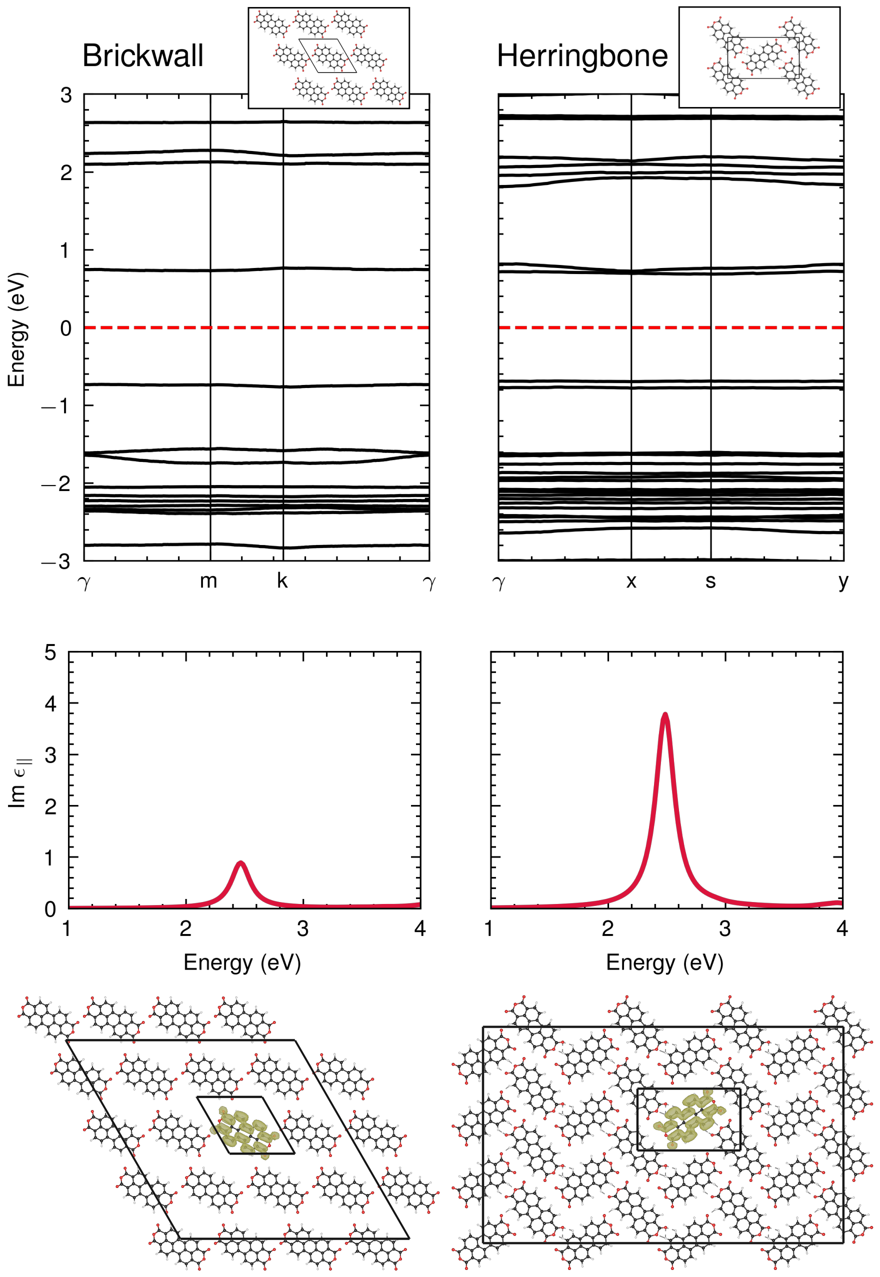

Supplementary Fig. 1. Comparison of the electronic and excitonic properties of a PTCDA monolayer in the brick-wall and herringbone geometry. Top: Kohn-Sham band structure of the brick-wall unit cell (left) and of the herringbone structure (right), both calculated with PBE. Middle: Imaginary part of the dielectric function obtained by the BSE. Bottom: Real-space representation of the respective lowest exciton state.
